# High-Throughput Sequencing Reveals Differential Expression of miRNAs in Intestine from Sea Cucumber during Aestivation

**DOI:** 10.1371/journal.pone.0076120

**Published:** 2013-10-15

**Authors:** Muyan Chen, Xiumei Zhang, Jianning Liu, Kenneth B. Storey

**Affiliations:** 1 Fisheries College, Ocean University of China, Qingdao, PR China; 2 LC-BIO CO., LTD. Hangzhou, PR China; 3 Institute of Biochemistry, Carleton University, Ottawa, Ontario, Canada; Auburn University, United States of America

## Abstract

The regulatory role of miRNA in gene expression is an emerging hot new topic in the control of hypometabolism. Sea cucumber aestivation is a complicated physiological process that includes obvious hypometabolism as evidenced by a decrease in the rates of oxygen consumption and ammonia nitrogen excretion, as well as a serious degeneration of the intestine into a very tiny filament. To determine whether miRNAs play regulatory roles in this process, the present study analyzed profiles of miRNA expression in the intestine of the sea cucumber (*Apostichopus japonicus*), using Solexa deep sequencing technology. We identified 308 sea cucumber miRNAs, including 18 novel miRNAs specific to sea cucumber. Animals sampled during deep aestivation (DA) after at least 15 days of continuous torpor, were compared with animals from a non-aestivation (NA) state (animals that had passed through aestivation and returned to the active state). We identified 42 differentially expressed miRNAs [RPM (reads per million) >10, |FC| (|fold change|) ≥1, FDR (false discovery rate) <0.01] during aestivation, which were validated by two other miRNA profiling methods: miRNA microarray and real-time PCR. Among the most prominent miRNA species, miR-200-3p, miR-2004, miR-2010, miR-22, miR-252a, miR-252a-3p and miR-92 were significantly over-expressed during deep aestivation compared with non-aestivation animals. Preliminary analyses of their putative target genes and GO analysis suggest that these miRNAs could play important roles in global transcriptional depression and cell differentiation during aestivation. High-throughput sequencing data and microarray data have been submitted to GEO database.

## Introduction

Aestivation is a fascinating phenomenon. Most research on aestivation has featured terrestrial or freshwater animals and is associated with environmental stressors such as high temperature and drought. To deal with these conditions, organisms descend into a hypometabolic state that allows long-term survival until environmental conditions permit a return to active life [1]–[5]. During long-term aestivation, animals reorganize cellular metabolism and reprioritize energy distribution to conserve metabolic fuels and suppress energy-expensive cellular processes [2]. The phenomenon of aestivation is less appreciated in the marine environment but recent studies with the sea cucumber, *Apostichopus japonicus* (Selenka, 1867), have shown that it is a good candidate model organism for studies of environmentally-induced aestivation by a marine invertebrate. This species has attracted more and more scientific attention, due to its phylogenetic position (an invertebrate deuterostome) and its special physiological characters. When sea water temperature rises above 25°C [6], sea cucumbers can be induced into aestivation and show a complete cessation of feeding and locomotor activities for more than 4 months [7]. During this period, obvious hypometabolism is observed, as evidenced by strong decreases in the rates of oxygen consumption and ammonia nitrogen excretion [6], [8], and degeneration of the intestine into a very tiny filament [7]. A return to active life is also observed when temperature is below about 18°C. Despite intense research that has been devoted to their physiological characteristics during aestivation [6], [8]–[10], little is known about the molecular level regulatory mechanisms of aestivation in sea cucumbers.

The role of microRNA (miRNA) in the control of hypometabolism is an emerging hot new topic. These small noncoding transcripts (∼22 nt long) are now recognized as key regulators of gene expression due to their influence on mRNA translation. By binding to target mRNA transcripts, miRNAs can reversibly inhibit translation of mRNAs and/or target them for degradation [11]. They are believed to regulate at least 60% and up to 90% of all mammalian mRNAs 12,13, but their involvement in hypometabolic regulation is only now beginning to be evaluated. Morin et al. [14] provided the first hint of a link between miRNA and hypometabolism by identifying nine miRNA species that were differentially expressed in tissues from non-hibernating and hibernating ground squirrels (*Spermophilus tridecemlineatus*). Following that, studies reported research linking hypometabolism and the reversible control of translation facilitated by miRNA action in anoxic turtles and freeze-tolerant frogs [15], [16]. Recently, stress-responsive alterations in the expression of miRNAs have been reported during natural freezing or anoxia exposure in an invertebrate species, the intertidal gastropod *Littorina littorea* [17]. Although this area of research is only beginning, the rapid and reversible characteristics of miRNA action in regulating the post-transcriptional expression of mRNA transcripts satisfy the need for the mechanisms of metabolic rate depression to be rapidly implemented and readily reversed once the situation is back to normal. This indicates that miRNAs may play a key role in achieving a hypometabolic state among stress-tolerant animals.

The presence of miRNAs across eukaryotic phylogeny and their striking sequence conservation among taxa has promoted the use of inter-specific high-throughput platforms for their analysis. Currently, the application of ultrahigh-throughput sequencing technologies such as Illumina/Solexa sequencing has greatly facilitated discovery and differential expression analysis of small RNAs. So far, only the global profile of small RNAs in a hibernating mammal has been described using this technology [18], but the present study demonstrates that it will be feasible to detect and study the involvement of miRNAs in hypometabolism regulation across different species, even those without full genome sequencing, such as the sea cucumber.

Herein, we present for the first time, using Solexa sequencing technology, an analysis of the global profile of small RNAs in sea cucumbers, comparing non-aestivation (NA) and deep aestivation (DA) states. We focus on intestine in the present study because it is the major site responsible for the strong metabolic rate depression seen under deep aestivating conditions and the global expression profile of mRNA transcripts from the this organ has also been constructed by applying RNA-seq technology in our previous study (Zhao and Chen, unpublished data). An analysis of the functional relevance of miRNA expression in relation to hypometabolism during aestivation is presented. A miRNA microarray and RT-qPCR were both used to supplement and confirm differentially expressed miRNAs. Our findings provide important new insights into the molecular mechanisms of sea cucumber aestivation.

## Results

### Small RNA library construction

**Figure 1:**
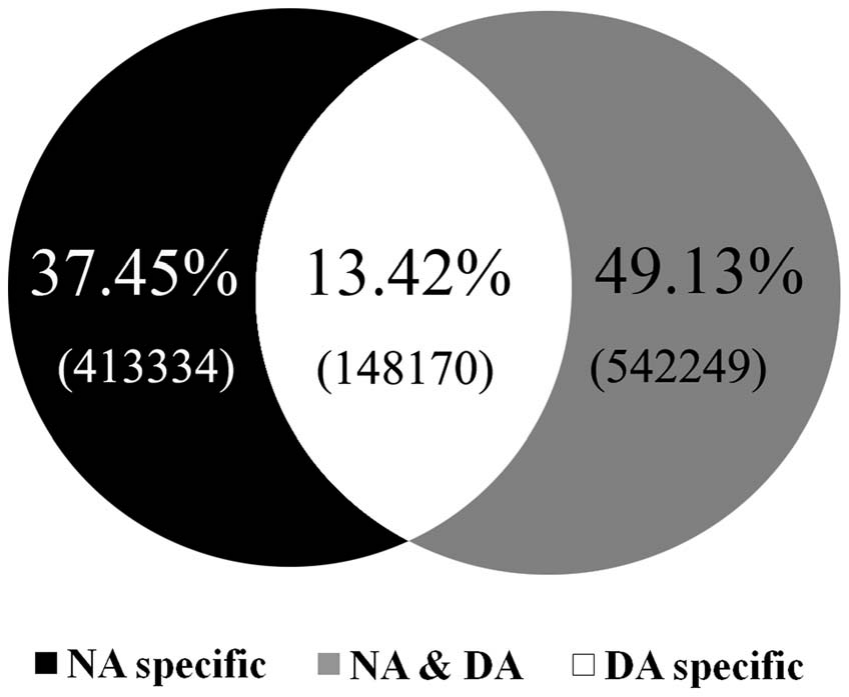
Common and specific sequences summary of unique sRNAs between non-aestivation (NA) and DA (deep-aestivation) states. Three groups are shown: those specific to NA, those specific to DA, and those that are common to both. The percentage distribution of sequences between these three groups is shown along with the number of sRNA sequences in brackets.

**Figure 2:**
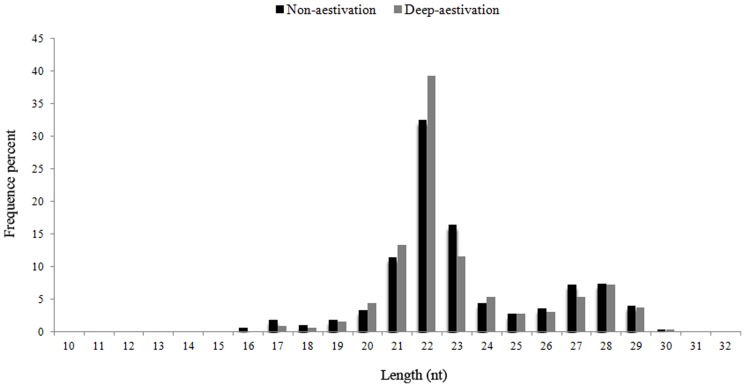
Length distributions of clean reads in NA and DA. The x-axis indicates sequence sizes from 10 nt to 32 nt. The y-axis indicates the percentage of reads for every given size.

In order to identify differentially expressed microRNAs during aestivation in sea cucumbers, we constructed small RNA libraries from intestines collected from two different physiological stages: deep aestivation (DA) and non-aestivation (NA). The criteria for judging DA status is that the intestine degenerates into a very tiny string (about 2∼3 mm), usually after 15 days of continuous torpor whereas animals in the NA state had passed through the aestivation period and returned to active status. High throughput Solexa sequencing of these two small RNA libraries yielded 10,876,248 (NA) and 11,194,928 (DA) total high quality clean reads (Table 1). Figure 1 shows that 1,103,753 unique sRNAs were also revealed, including 148,170 common reads, and 413,334 and 542,249 specific reads for NA and DA, respectively. Of these, a total of 21557 (NA) and 26862 (DA) unique small RNAs were identified as either rRNA (17211 for NA and 20960 for DA), snRNA (314 for NA and 416 for DA), snoRNA (43 for NA and 43 for DA), or tRNA (3989 for NA and 5443 for DA) against the NCBI Genebank and Rfram 10.1 database using BLAST searches (Table 1). The histograms of the corresponding length distributions of reads show similar trends between these two libraries by following a typical distribution pattern, and the majority of sequences were 22 nt in length (Fig. 2).

**Table 1:** Mapping statics of Solexa sequencing reads.

	Unique sRNAs	Percent (%)	Total sRNAs	Percent (%)
	(NA vs DA)	(NA vs DA)	(NA vs DA)	(NA vs DA)
Total number of reads	561504/690419	100%/100%	10876248/11194928	100%/100%
Reads mapped to:				
miRNAs	14687/20110	2.62%/2.91%	6782785/6715567	62.36%/59.99%
rRNAs	17211/20960	3.07%/3.04%	162583/110189	1.49%/0.98%
snRNAs	314/416	0.06%/0.06%	762/1368	0.01%/0.01%
snoRNAs	43/43	0.01%/0.01%	90/74	0%/0%
tRNAs	3989/5443	0.71%/0.79%	27147/125660	0.25%/1.12%
unann	525260/643447	93.55%/93.2%	3902881/4242070	35.88%/37.89%

### Identification of conserved miRNAs

The small RNAs were searched against the latest miRBase release version 19.0 (miRBase V19.0) to identify conserved miRNAs in sea cucumber intestine. A total of 14687 and 20110 unique miRNAs were searched in the NA and DA libraries, respectively. For each miRNA, the highest expression miRNA for each mature miRNA family was chosen as the reference sequence for constructing the temporary miRNA database. In total, 252 and 263 conserved sequences were annotated for NA and DA, respectively, as sea cucumber miRNAs. Among them, 225 were classified as common miRNAs, 38 as specific miRNAs in the NA library, and 27 as specific miRNAs in the DA library. All conserved miRNAs detected by sequencing with RPM (reads per million) >10 are listed in Table S1.

### Identification and validation of novel miRNA candidates

After filtering these data sets and identifying conserved miRNAs, the 525260 (NA) and 643447 (DA) unannotated reads were analyzed to predict novel miRNAs using Mireap software by exploring the secondary structure with a prediction strategy as follows: (1) the sRNAs which matched to the *S. purpuratus* genome and *A. japonicus* transcriptome and ESTs (expressed sequence tags) were selected; (2) hairpin miRNAs can fold secondary structures and mature miRNAs are present in one arm of the hairpin precursors; these, were considered as candidate miRNA genes; (3) the secondary structures of the hairpins are steady, with the free energy of hybridization lower than or equal to -18 kcal/mol. Furthermore, RT-PCR was adopted to validate our sequencing data and all primers were listed in Table S2. A total of 18 novel miRNAs were validated in intestine by RT-PCR (Fig. S1).

### Different expression profiles of identified miRNAs between NA and DA samples

The expression of known miRNAs in these two samples was demonstrated by calculating Log2-ratio and plotting as a Scatter Plot. The scatter plot was modified to show a value of 0.01 in cases of the absence of a miRNA expression. The most abundant miRNAs in the Solexa sequence data were miR-10a (2973660 in NA and 1482171 in DA) and miR-10a-5p (2891589 in NA and 1423173 in DA) from the same miRNA family. These accounted for 65% of the total Solexa sequence reads mapped to all miRNAs. To reliably quantify miRNA expression, we restricted our analysis to differentially expressed miRNAs with the following three criteria: RPM>10, |FC|≥1 and FDR<0.01. Based on these criteria, 42 differentially expressed miRNAs with 28 up-regulated and 14 down-regulated miRNAs were found between NA and DA stages (Table 2).

**Table 2:** Differentially expressed miRNAs in sea cucumber intestine between NA and DA states as detected with Solexa sequencing and microarray analysis.

	microRNA sequencing					microarray				
miRNA	RPM (NA)	RPM (DA)	FC_Log2 Ratio (DA/NA)	P-value	FDR	Mean signal (NA)	Mean signal (DA)	FC_Log2 Ratio (DA/NA)	P-value	FDR
miR-200-3p*	1011	3148	1.60	2.80E-239	1.10E-238	1359	6563	2.27	6.26E-05	2.08E-04
miR-2004*	2633	7067	1.38	0	0	19984	49699	1.31	1.70E-05	6.88E-05
miR-2010*	3564	14243	1.96	0	0	659	5803	3.14	4.73E-05	1.65E-04
miR-22*	181	736	1.98	9.39E-77	3.05E-76	135	641	2.25	1.15E-05	5.04E-05
miR-252a*	114	355	1.60	1.59E-28	3.87E-28	104	591	2.51	2.46E-08	2.31E-07
miR-252a-5p*	116	357	1.58	2.91E-28	6.99E-28	103	593	2.53	6.14E-09	1.05E-07
miR-7*	10559	29640	1.45	0	0	440	4101	3.22	2.04E-05	7.78E-05
miR-92*	55629	142056	1.31	0	0	4758	9777	1.04	2.09E-10	4.31E-08
miR-92a*	43473	90546	1.02	0	0	4217	8821	1.06	2.45E-09	8.41E-08
miR-153	58	299	2.32	5.77E-39	1.58E-38	5378	4439	-0.28	4.50E-04	1.10E-03
miR-153-3p	51	268	2.35	1.45E-35	3.76E-35	4662	2884	-0.68	5.65E-06	2.64E-05
miR-2008	405	1618	1.96	9.83E-164	3.60E-163	9953	1092	-3.19	2.92E-09	8.60E-08
miR-310	33	184	2.44	6.74E-26	1.52E-25	483	823	0.77	1.77E-06	9.84E-06
miR-310-3p	25	84	1.71	1.86E-08	2.94E-08	1670	1114	-0.58	9.99E-03	1.38E-02
miR-71a	153	410	1.38	1.18E-26	2.71E-26	1798	1550	-0.21	4.81E-02	5.32E-02
miR-71a-5p	25	84	1.71	1.86E-08	2.91E-08	8982	9585	0.09	9.73E-02	9.82E-02
miR-92c	41719	85216	0.9888	0	0	4241	8728	1.04	3.35E-10	3.45E-08
miR-92-3p	40066	81951	0.9907	0	0	4146	8610	1.05	1.15E-09	5.91E-08
miR-235a	6071	10122	0.6958	4.89E-200	1.80E-199	4720	9771	1.05	7.50E-08	5.72E-07
miR-7-5p	13133	23804	0.8163	0	0	454	4611	3.34	1.56E-05	6.57E-05
miR-2012-5p	475339	804653	0.7177	0	0	3150	6703	1.09	6.06E-05	2.05E-04
miR-2008	405	1618	1.9566	9.83E-164	3.60E-163	9953	1092	-3.19	2.92E-09	8.60E-08
let-7a-5p	817	1658	0.9794	3.74E-60	1.14E-59	4284	1488	-1.53	1.88E-05	7.45E-05
miR-210	91179	86593	-0.116	1.48E-64	4.62E-64	13777	2116	-2.70	1.34E-04	4.12E-04
miR-210-3p	93148	88209	-0.12	1.80E-70	5.74E-70	13782	2108	-2.71	1.41E-04	4.28E-04
let-7-5p	996	1456	0.5061	7.83E-18	1.63E-17	645	107	-2.59	1.77E-04	5.00E-04
miR-31a-5p	2181	1517	-0.565	2.97E-32	7.35E-32	11587	4989	-1.22	6.09E-04	1.38E-03
miR-2011	290828	305417	0.0289	9.51E-15	1.83E-14	57203	14562	-1.97	8.94E-04	1.96E-03
miR-375-3p	90125	119762	0.3685	0	0	613	101	-2.60	1.05E-03	2.21E-03
miR-31-5p	209383	168625	-0.354	0	0	11128	5180	-1.10	1.14E-03	2.36E-03
miR-72-5p	191131	158706	-0.31	0	0	10172	4848	-1.07	1.27E-03	2.57E-03
miR-375	96422	129304	0.3817	0	0	646	101	-2.68	1.91E-03	3.36E-03
miR-29a	2032	3541	0.7596	1.01E-82	3.35E-82	1621	342	-2.25	2.89E-03	4.76E-03
miR-375b-3p	68871	90878	0.3584	0	0	673	124	-2.44	3.05E-03	4.94E-03
miR-10a-5p	2891589	1423173	-1.06	0	0	27597	26595	-0.05	1.66E-03	3.02E-03
miR-2006	6401	1762	-1.90	0	0	4292	2835	-0.6	4.05E-05	1.49E-04

NOTE: miRNAs with RPM>10 or Signal>500, FDR<0.01 are shown. “*”: designates miRNAs that are differentially expressed in the same pattern using both Solexa sequencing and microarray analysis with |FC|≥1.0.

### Validation of differential expression of miRNAs by microarray and real-time PCR

We used the LC Science microarray platform as an independent miRNA profiling method to search for additional differentially expressed miRNAs. The microarray detected 290 miRNA candidates in at least one of the two stages; 32 miRNAs were significantly differentially expressed between NA and DA using Signal>500, |FC|≥1 and FDR<0.01 as the criterion (Table 2). There were 9 miRNAs identified as significantly differentially expressed miRNAs by both Solexa sequencing and miRNA microarray methods (Table 2). All of them showed an over-expressed pattern during DA compared to NA stage as assessed by both technologies.

**Figure 3:**
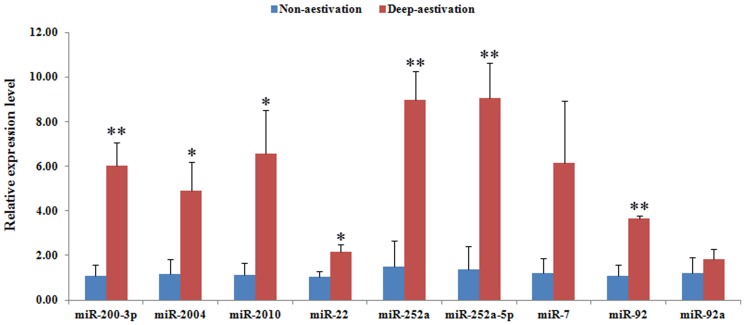
Real-time PCR validation of differentially expressed miRNAs supported by both Solexa sequencing and microarray. NA, non-aestivation; DA, deep-aestivation (n = 3 for NA and DA respectively). The RT-PCR was performed in triplicate wells for each individual sample. Bars show means ± standard deviation. “*” p<0.05; “**” p<0.01.

For validation and identification of the aestivation-related miRNAs in the sea cucumber, RT-PCR analysis of the 9 differentially expressed miRNAs detected by both Solexa sequencing and miRNA microarray was performed. As illustrated in Figure 3, all of these miRNAs showed a consistent expression pattern with the results from Solexa sequencing and microarray. Among them, miR-200-3p, miR-2004, miR-2010, miR-22, miR-252a, miR-252a-5p and miR-92 were significantly over-expressed in DA by amounts ranging from 2- to 9-fold greater than NA values.

### Gene ontology

To better understand the functions of miRNAs, putative targets of the 9 up-regulated miRNAs were predicted by applying the forecasting software TargetScan based on a recent sequencing of the *A. japonicus* transcriptome [19]. Then GO analysis was used to identify enriched functional groups (FDR<0.05) among miRNA putative targets (Table S3); the putative target genes involved in enriched functional groups are listed in Table S4. Transcription termination, DNA-dependent, regulation of ATPase activity and generation of precursor metabolites and energy (FDR<0.05) were among the most significant biological processes related to aestivation.

## Discussion

Since the first study indicating a relationship between miRNA regulation and hypometabolism among stress-tolerant animals [14], the emerging multiple roles of miRNAs in the molecular responses of metabolic rate depression have been reviewed by Biggar and Storey [20], drawing links to miRNA regulation with mitogen-activated protein kinase (MAPK) signaling, translational arrest, reduced muscle wasting, cell cycle arrest and anti-apoptotic signaling. Based on that, we hypothesized that global gene transcriptional depression may be partly regulated by miRNAs during sea cucumber aestivation. Our current study, using three different technologies, reports several differentially expressed miRNAs, which provides support to our hypothesis.

In the present study, over half of the sequences in NA (60.5%) and DA (64.23%) had sizes between 21–23 nt, which is consistent with typical miRNA sizes and similar sizes of miRNAs in the sea urchin *S. purpuratus* [21]. For further annotation, the genome of the purple sea urchin (*Strongylocentrotus purpuratus*) and the transcriptome of the sea cucumber *A. japonicus* were used as references for sRNA mapping. *S. purpuratus* and *A. japonicus* both belong to the Echinodermata and *S. purpuratus* is the closest species to *A. japonicus* that has a sequenced genome. The recently published transcriptome of *A. japonicus* is currently the most comprehensive resource for this species [19]. As shown in Table 1, 2.62% (NA) and 2.91% (DA) of unique sRNAs were annotated as miRNAs. This small mapping proportion and a large number of unannotated sRNAs (Table 1) are due to the genome differences among species, limited transcriptome information, and sequencing errors. Considering the lack of genome background and limited transcriptome information for sea cucumber, no further annotation was conducted for this species. The most abundant miRNAs were miR-10a and miR-10a-5p, accounting for 86% and 43% of miRNA reads in NA and DA, respectively, which is different from the results reported by Li et al. [22]. They identified miR-10 as the most abundant miRNA in sea cucumber haemocytes (we found that miR-10 expression was very low in intestine). Together the results of these two studies suggest tissue-specific differences in miRNA abundance in *S. purpuratus*. It has been reported that miR-10a is involved in apoptosis [23], protein synthesis [24], embryo development and differentiation [25], [26], hematopoietic stem cell mobilization [27] and inflammation [28], suggesting the potential for multiple functions of miR-10a in aestivation.

Three independent technologies were applied to construct the miRNA profile and to authenticate each other. All three methods have their own merits and drawbacks [29]. The development of an ultrahigh-throughput sequencing RNA-Seq technique such as Illumina/Solexa, provided a powerful technology that greatly facilitated discovery and analysis of small RNAs, especially for an animal like the sea cucumber for which there is no genome background and no miRNA data in miRbase. This technology can generate several million small RNA sequences in each small RNA library in one run and has been successfully and increasingly used for non-model species like the sea star, *Patiria miniata* [21]. The RNA-Seq technique has the potential to overcome microarray limitations (lower throughput, high background noise, lower sensitivity and the miRNA sequence has to be known) and provide an expression profile with a greater and reproducible dynamic range. However this method can be influenced by several factors like sequencing errors and raw data processing, and has to be complemented and supported by more traditional methods including microarray and qPCR, the “gold standard” in detecting and quantifying gene expression. Our results identified very limited numbers of significantly differentially expressed miRNAs by both Solexa sequencing and microarray technologies, which is consistent with the findings in hibernating ground squirrels [18]. Moreover, these differentially expressed miRNAs supported by both technologies have a high success rate in stem-loop qPCR validation.

Profiling of miRNA expression showed that miR-22, miR-92a, miR-92, miR-7, miR-252a, miR-252a-5p, miR-2004, miR-2010 and miR-200-3p were specifically expressed in the intestine of sea cucumber during aestivation. MiR-22 has been connected with multiple biological processes including tumorigenesis, epigenetic modification, embryonic development, and skeletal muscle metabolism [30]. MirR-22 is also involved in a regulatory loop with PTEN and AKT [31] and has been shown to act as both a positive and negative regulator of PTEN expression [32], [33]. In our study, miR-22 was significantly up-regulated in the intestine of aestivating sea cucumber compared to non-aestivating controls. It has been reported that the PI3/Akt pathway is one of the major pathways regulating hypometabolism [34], [35] and PTEN is the primary protein phosphatase acting to suppress this signal by removing phosphate groups from members of the pathway [36]. Enhanced levels of miR-22 in the intestine suggest that miR-22 may act as a mechanism to dampen PI3-K/Akt signaling during metabolic rate depression to control protein translation. MiR-92a and miR-92, identified as part of the miR-17-92 cluster, have been linked with anti-apoptotic properties in colon cancer [37] and play a role in cell proliferation. Significant up-regulation of miR-92 was also detected in the intestine of deep aestivating sea cucumbers, which suggests that this miRNA may act to arrest the apoptotic response and help to maintain homeostasis under energy limited conditions like aestivation. Recently, miR-7 has been shown to have functional roles in the differentiation of intestinal epithelial cells [38] and plays an important role in developmental decision-making in association with signal-transduction pathways [39]. Sea cucumber intestine also showed a significant increase in miR-7 expression levels during aestivation and could be involved in the suppression of growth and proliferation in response to global metabolic rate depression during torpor. Although the physiological functions of miR-252a, miR-252a-5p, miR-2004, miR-2010 and miR-200-3p are still not known, their specific expression patterns indicate that they are also likely to play a role in the hypometabolic process in sea cucumber intestine. Further experiments are needed to elucidate their roles in this process.

To gain insight into the potential functions of the differentially expressed miRNAs during aestivation, we predicted the putative targets of these miRNAs using Targetscan. Then, Gene Ontology analysis classified the potential enriched functional groups (Table S3). The potential network of miRNAs and genes that are involved in hypometabolism during aestivation seems to be highly complicated. Nevertheless, a special focus was given to certain highly enriched functions related to torpor. A significant function of the differentially expressed miRNA targets is transcription termination. This is linked to the fact that global transcriptional depression during torpor is one of the most important strategies that promotes long-term viability in the hypometabolic state [2], which is also observed in aestivating sea cucumbers (unpublished data).

## Materials and Methods

### Ethics Statement

Not applicable. Our research did not involve human and vertebrate species or samples. No permission was needed for sea cucumber collection. The sea cucumber (*A. japonicus*) is not an endangered or protected species.

### Animals

Sea cucumbers (two years old; body weight 70–80 g) were collected from culture ponds in Jiaozhou Bay of the Yellow Sea in China. Animals sampled in April (sea water temperature about 15°C) have gone through the aestivation period, regenerated their tissue and returned to active status; these were used as non-aestivating controls. The deep aestivating animals were collected in August (sea water temperature above 25°C); the animals had stopped feeding and locomotion and the intestine had degenerated into a very tiny string (about 2∼3 mm), usually after 15 days of continuous torpor. The intestine of each animal was dissected without contents and immediately frozen in liquid nitrogen, then kept at −80°C until subsequent analysis.

### Small RNA library preparation and sequencing

Digestive tissue samples from 12 sea cucumbers were used in this study including 6 non-aestivating animals (NA) and 6 deep aestivating animals (DA). Total RNA from each tissue sample was extracted using miRNeasy Mini kit (Qiagen) according to the manufacturer's instructions. Total RNA quality was checked with a Bioanalyzer 2100 (Agilent Technologies). Total RNA of sea cucumbers from the same stage were mixed in equal amounts into two pooled samples: NA and DA. The overall flow of the sequencing procedure for small RNA is shown in Figure S2. Briefly, the population of recovered small-RNAs, ranging from 18 to 30nt in length was purified from 15% polyacrylamide gels, and these small RNAs were then ligated sequentially to 5′ and 3′ adapters. Reverse transcription was performed followed by PCR amplification. The purified PCR products were used directly for cluster generation and sequencing analysis using the Illumina's Solexa Sequencer according to the manufacturer's instructions (BGI, China). Then the image files generated by the sequencer were processed to produce digital-quality data.

### Sequence data analysis

The raw reads obtained from Solexa sequencing were processed by trimming poor quality reads, 5′ adapter pollution reads, reads without 3′ adapters, reads without insert fragments, reads containing poly(A) stretches, and reads less than 18 nt. Other RNAs (rRNA, tRNA, snRNA and snoRNA) were removed by blasting against the GenBank database (http://blast.ncbi.nlm.nih.gov) and the Rfram database (http://sanger.ac.uk/software/Rfam). Considering that no miRNA information for the sea cucumber was in miRBase19.0, the remaining clean reads were aligned to search all known precursor/mature miRNAs of all animal species in miRBase 19.0 for perfect matches. We then chose the highest expression miRNA for each mature miRNA family which was regarded as a temporary miRNA database. Clean data were aligned to the above temporary miRNA database and the expression of miRNA is generated by summing the count of reads which can align to the temporary miRNA database without mismatches. Finally, we predicted the precursor of the identified miRNAs; those that could not fold into a hairpin structure were regarded as pseudo-miRNA. After removing the conserved miRNA sequences, the remaining small RNA sequences were used to perform Blastn searches against the *S. purpuratus* genome and *A. japonicus* transcriptome to obtain precursor sequences for novel potential miRNAs. Potentially novel miRNAs were identified using MIREAP (http://sourceforge.net/projects/mireap/) with stem-loop structure prediction.

For comparing the expression of known and novel miRNAs between the two samples (NA and DA), plotting on a Log2-ratio figure and Scatter Plot were applied to show differential expression levels. The normalizing procedures followed the BGI standard protocol: (1) Normalize the expression of miRNA in two samples (NA and DA) to get the expression of reads per million (RPM). The Normalized expression  =  Actual miRNA count/Total count of clean reads *1000000; (2) Calculate fold-change and P-value from the normalized expression. Then generate the log_2_ ratio plot and scatter plot. Fold change  =  log_2_(DA/NA) and P-value forum is shown as below:
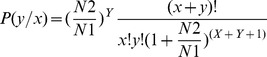



In this forum, the total clean tag number of NA is defined as N1, and the total clean tag number of DA as N2; miRNA A holds x tags in NA and y tags in the DA library. The FDR (False Discovery Rate) was also estimated, to determine the threshold of P-value. The "FDR<0.01 and the absolute value of log_2_ (DA/NA)≥1.0" was used as the criterion to judge the significance of miRNA expression difference. If the normalized expression was zero, it was changed to 0.01; some miRNAs showing a ratio of less than 1 in both samples were not analyzed in the differential analysis.

### miRNA microarray and data analysis

Total RNA (including miRNA) was collected from the samples as described above. After confirming their integrity, the total RNA was sent to LC Sciences (Hangzhou, China) to be processed using their miRNA Microarray Service. Each chip produced contained probes for 341 sea cucumber miRNAs from Solexa sequencing, 546 sea squirt *Ciona intestinalis* miRNAs, 101 acorn worm *Saccoglossus kowalevskii* miRNAs and 50 sea urchin *S. purpuratus* miRNAs from miRBase v 19.0. Chip hybridization experiments were carried out in triplicate from different biological samples. Labeling and hybridization of total RNA were performed according to the protocol from LC Sciences for a single-color experiment with no modification. After hybridization, signals were detected using tag-specific Cy3 dye. Hybridization images were collected using a laser scanner (GenePix 4000B, Molecular Device) and digitized using Array-Pro image analysis software (Media Cybernetics). Microarray data were deposited in a database (ArrayExpress, GEO) with accession number GSE48869. Data were analyzed by first subtracting the background, then the signals were normalized using a Lowess (locally weighted regression) filter. The raw microarray data set was filtered according to a standard procedure to exclude spots with minimum intensity. It was arbitrarily set to an intensity parameter of P100 for the miRNA microarray data. Spots with diameters less than 10 µm and flagged spots were also excluded from the analyses. Statistical tests were also performed by LC Sciences following their own standardized procedures. To identify DA induced statistically significance for differentially expressed miRNAs, a criterion of signal >500, |fold change|≥1.0 and FDR <0.01 was used.

### Quantitative miRNA real-time PCR assay

For miRNA analyses 100 ng total RNA were reverse-transcribed with miRNA-specific stem-loop RT primers and Reverse Transcriptase M-MLV (RNase H^−^) (Takara, Japan) according to the experimental protocol. The stem-loop reverse transcription primers were designed following the method described by Chen et al. (2005) [40]. The reaction proceeded for 60 min at 42°C, followed by 15 min at 70°C and hold at 4°C. Following RT, the cDNA was amplified by real-time PCR using platinum SYBR Green qPCR SuperMix-UDG (Invitrogen, USA) with miRNA specific forward and reverse primers. As an internal control to normalize for technical variations, 5.8s rRNA was also amplified. All reactions were performed on 3 biological replicates, each of them being run three times. Negative controls containing all reagents except template were included on each reaction plate. All primers for RT-PCR and qPCR are listed in supplementary Table S5. The relative expression (fold changes) of miRNA was calculated using the 2^−△△^ method, and the level of significance was analyzed by one-way analysis of variance (ANOVA) with SPSS statistics 18.0.

### miRNA target prediction and GO enrichment analysis

We tried to extract sea cucumber 3′ untranslated regions (UTRs) based on the sea cucumber transcriptomic database [19]. The position of 2–8 nt in a mature miRNA is called the seed region which is highly conserved, and this seed region most often binds to a target site in the 3′ UTR of the target mRNA by perfect complementarity. The target genes of miRNAs were predicted by the TargetScan algorithm complying with one of the following criteria in the seed region: (1) no mismatch between 2–7 nt on the 5′ end of miRNA and the first position in the 3′ UTR of the target mRNA is A (7mer-1a); (2) no mismatch between 2–8 nt on the 5′ end of miRNA (7mer-m8); (3) no mismatch between 2–8 nt on the 5′ end of miRNA and the first position in the 3′ UTR of the target mRNA is A (8mer). Due to imperfect miRNA-target interaction in animals and a lack of full-length mRNAs and genomic background available for sea cucumbers, no other criteria were defined.

GO (Gene Ontology) analysis was performed for the target genes of both confirmed differentially expressed miRNAs applying Solexa sequencing and microarray, mapping them to GO terms in the database (http://www.geneontology.org/). GO analysis identified enriched functional groups (false discovery rate [FDR]<0.05) among miRNA putative targets.

## Supporting Information

Figure S1 **18 novel miRNAs were validated in sea cucumber intestine by RT- PCR. NTC: no template control; Lanes are as follows.** A: 1. 50Marker, 2. novel-miR-1_NA, 3. novel-miR-1_DA, 4. novel-miR-1_NTC, 5. novel-miR-11_NA, 6. novel-miR-11_DA, 7. novel-miR-11_NTC, 8. novel-miR-12_NA, 9. novel-miR-12_DA, 10. novel-miR-12_NTC, 11. novel-miR-13_NA, 12. novel-miR-13_DA, 13. novel-miR-13_NTC, 14. novel-miR-14_NA, 15. novel-miR-14_DA, 16. novel-miR-14_NTC; B: 1. 50Marker, 2. novel-miR-15_NA, 3. novel-miR-15_DA, 4. novel-miR-15_NTC, 5. novel-miR-16_NA, 6. novel-miR-16_DA, 7. novel-miR-16_NTC, 8. novel-miR-17_NA, 9. novel-miR-17_DA, 10. novel-miR-17_NTC, 11. novel-miR-18_NA, 12. novel-miR-18_DA, 13. novel-miR-18_NTC, 14. novel-miR-19_NA, 15. novel-miR-19_DA, 16. novel-miR-19_NTC; C: 1. 50Marker, 2. novel-miR-20_NA, 3. novel-miR-20_DA, 4. novel-miR-20_NTC, 5. novel-miR-21_NA, 7. novel-miR-21_DA, 8. novel-miR-21_NTC; 9. novel-miR-22_NA, 10. novel-miR-22_DA, 11. novel-miR-22_NTC, 12. novel-miR-23_NA, 13. novel-miR-23_DA, 14. novel-miR-23_NTC, 15. novel-miR-24_NA, 16. novel-miR-24_DA, 17. novel-miR-24_NTC D: 1. 50Marker, 2. novel-miR-25_NA, 3. novel-miR-25_DA, 4. novel-miR-25_NTC, 5. novel-miR-26_NA, 6. novel-miR-26_DA, 7. novel-miR-26_NTC, 8. novel-miR-27_NA, 9. novel-miR-27_DA, 10. novel-miR-27_NTC.
(TIF)

Figure S2 **Overall flow chart of the sequencing procedure for small RNA.**
(TIF)

Table S1 **All conserved miRNAs detected by sequencing with RPM (reads per million) >10 from either NA or DA.**
(XLSX)

Table S2 **Primers used in novel miRNAs validation.**
(XLSX)

Table S3 **Enriched GO categories of the predicted targets of 9 up-regulated miRNAs during aestivation with** ***FDR*** **<0.05.**
(XLSX)

Table S4 **List of putative targets involved in enriched GO categories.**
(XLSX)

Table S5 **Primers used in the stem-loop RT-qPCR experiment.**
(XLSX)
