# Supplementary figures and images for: High-Throughput Sequencing Reveals Differential Expression of miRNAs in Intestine from Sea Cucumber during Aestivation
Source: PLoS One. 2013 Oct 15;8(10):e76120. doi: 10.1371/journal.pone.0076120 (PMC3797095; doi:10.1371/journal.pone.0076120)

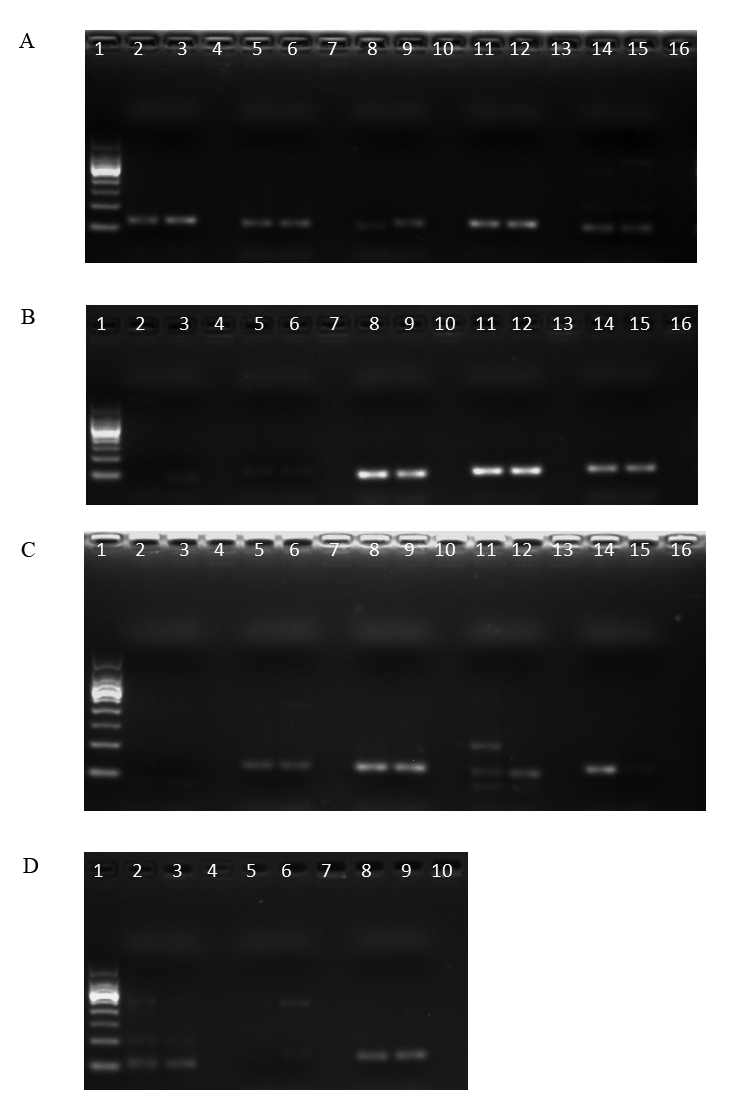

Supplement: Figure S1 — 18 novel miRNAs were validated in sea cucumber intestine by RT- PCR. NTC: no template control; Lanes are as follows. A: 1. 50Marker, 2. novel-miR-1_NA, 3. novel-miR-1_DA, 4. novel-miR-1_NTC, 5. novel-miR-11_NA, 6. novel-miR-11_DA, 7. novel-miR-11_NTC, 8. novel-miR-12_NA, 9. novel-miR-12_DA, 10. novel-miR-12_NTC, 11. novel-miR-13_NA, 12. novel-miR-13_DA, 13. novel-miR-13_NTC, 14. novel-miR-14_NA, 15. novel-miR-14_DA, 16. novel-miR-14_NTC; B: 1. 50Marker, 2. novel-miR-15_NA, 3. novel-miR-15_DA, 4. novel-miR-15_NTC, 5. novel-miR-16_NA, 6. novel-miR-16_DA, 7. novel-miR-16_NTC, 8. novel-miR-17_NA, 9. novel-miR-17_DA, 10. novel-miR-17_NTC, 11. novel-miR-18_NA, 12. novel-miR-18_DA, 13. novel-miR-18_NTC, 14. novel-miR-19_NA, 15. novel-miR-19_DA, 16. novel-miR-19_NTC; C: 1. 50Marker, 2. novel-miR-20_NA, 3. novel-miR-20_DA, 4. novel-miR-20_NTC, 5. novel-miR-21_NA, 7. novel-miR-21_DA, 8. novel-miR-21_NTC; 9. novel-miR-22_NA, 10. novel-miR-22_DA, 11. novel-miR-22_NTC, 12. novel-miR-23_NA, 13. novel-miR-23_DA, 14. novel-miR-23_NTC, 15. novel-miR-24_NA, 16. novel-miR-24_DA, 17. novel-miR-24_NTC D: 1. 50Marker, 2. novel-miR-25_NA, 3. novel-miR-25_DA, 4. novel-miR-25_NTC, 5. novel-miR-26_NA, 6. novel-miR-26_DA, 7. novel-miR-26_NTC, 8. novel-miR-27_NA, 9. novel-miR-27_DA, 10. novel-miR-27_NTC. (TIF) [file pone.0076120.s001.tif]

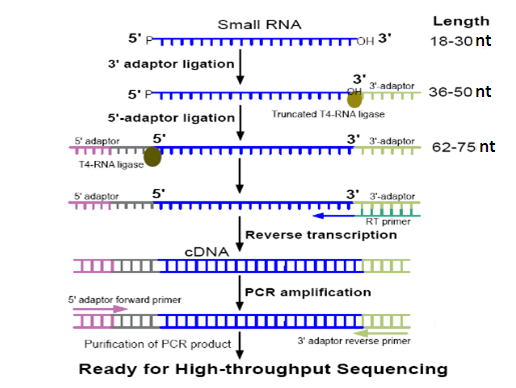

Supplement: Figure S2 — Overall flow chart of the sequencing procedure for small RNA. (TIF) [file pone.0076120.s002.tif]
